# Impact of Thiazides and Fluoropyrimidines Interaction on Myelotoxicity and Other Adverse Events in Real‐World Practice: A Retrospective Cohort Study

**DOI:** 10.1002/cam4.71650

**Published:** 2026-02-26

**Authors:** Gerard Ronda‐Roca, José Porcel‐Maleno, Mónica Lobo‐Palomar, Gustavo Centeno‐Soto, Ana Ruiz‐Casado, Belén Ruiz‐Antorán

**Affiliations:** ^1^ Department of Clinical Pharmacology Hospital Universitario Puerta de Hierro‐Majadahonda. Majadahonda Madrid Spain; ^2^ Department of Medical Oncology Hospital Universitario Puerta de Hierro‐Majadahonda. Majadahonda Madrid Spain

**Keywords:** capecitabine, colorectal cancer, drug interaction, fluoropyrimidines, myelotoxicity, oxaliplatin, thiazides

## Abstract

**Introduction:**

The European Summary of Product Characteristics (SmPC) for 5‐FU warns of significant granulocyte decline when combined with thiazides, cyclophosphamide, or methotrexate, based on a 1981 cohort of 14 patients. Despite limited evidence, drug‐interaction checkers still flag this risk. We conducted a retrospective cohort study to compare myelotoxicity in patients receiving fluoropyrimidines with or without thiazides. Secondary objectives included assessing clinical relevance and overall safety.

**Methods:**

A retrospective, single‐center observational study was performed using electronic health records. The cohort included colorectal cancer patients treated with capecitabine or capecitabine‐oxaliplatin, with a maximum of one prior chemotherapy line. Myelotoxicity was defined using local laboratory thresholds. Clinical relevance was assessed through predefined adverse events (AEs). All AEs and lab data were collected up to 1 month after chemotherapy completion.

**Results:**

We included 192 patients (mean age 68.6 ± 13 years; 61.5% male); 37 (19.3%) were on thiazides at treatment start. Baseline characteristics were largely comparable between groups, although thiazide users were older. Median follow‐up was 126 days (IQR: 84–158). Hemoglobin declined significantly in thiazide users at 1–3 months (−0.3 vs. +0.11 g/dL; *p* = 0.006) and > 6 months (−2.63 vs. −0.75 g/dL; *p* = 0.002). Other hematologic parameters showed no significant differences. Myelotoxicity occurred in 83.8% of thiazide users vs. 75.5% in controls (RR = 1.11; 95% CI: 0.94–1.31; *p* = 0.280). No differences were seen in febrile neutropenia, mucositis, infections, or other AEs. Logistic regression showed no association between thiazide use and myelotoxicity risk.

**Conclusions:**

Concomitant use of fluoropyrimidines and thiazides was not associated with increased myelotoxicity or clinically relevant adverse events. These findings suggest that the clinical impact of this interaction may be lower than currently assumed and should be confirmed in larger multicenter studies.

**Trail Registration:**

The protocol was submitted and approved at the Spanish Clinical Studies Registry (REEC ID: 0050‐2024‐OBS) and the EMA‐RWD Catalogs (ID: 1000000181).

## 1 Introduction

Fluoropyrimidines are pyrimidine analogs indicated in gastrointestinal tumors, metastatic breast cancer, and advanced head and neck tumors [1, 2, 3]. This drug class includes 5‐fluorouracil (5‐FU), capecitabine, and tegafur. 5‐FU is administered intravenously while tegafur and capecitabine (5‐FU prodrug) are administered orally [1, 2, 3]. Genotyping dihydropyridine dehydrogenase (DPYD) before prescribing fluoropyrimidines is essential, since it guides dosing and reduces toxicity risk [4, 5]. Common adverse events (AEs) are gastrointestinal epithelial damage, myelotoxicity, electrocardiographic changes, infections, palmar‐plantar erythrodysesthesia, and alopecia [1, 2, 3, 6].

Thiazides diuretics exert their function through the inhibition of sodium and chloride transporters at the distal convoluted tubule, promoting natriuresis [7]. Among these, there are pure thiazides such as hydrochlorothiazide and bendroflumethiazide, and thiazide‐related drugs (chlortalidone and indapamide). Their main indication is arterial hypertension, but other indications are insipid central diabetes and idiopathic hypercalciuria [8]. Frequent AEs are electrolytic imbalance, kidney function deterioration, or glucose intolerance [7, 8]. Leucopenia and agranulocytosis are rare and occur in ≤ 1/10,000 patients [8].

Cancer patients are at higher risk of drug interactions and those may lead to a higher risk of AEs, especially when kidney function is altered [9, 10]. The European 5‐FU summary of product characteristics (SmPC) states that in patients treated with cyclophosphamide, methotrexate, and 5‐FU, the addition of hydrochlorothiazide caused a marked decrease of granulocytes in comparison with those who did not receive hydrochlorothiazide [1]. This comes from a 1981 cohort of 14 breast cancer patients that showed a higher risk of agranulocytosis when treated with thiazides and chemotherapy regimens that contained 5‐FU [11]. Hydrochlorothiazide's SmPC specifies that it may reduce renal excretion of cytotoxic drugs and therefore strengthen their myelosuppressive effects [8]. In capecitabine and tegafur SmPCs this interaction is not stated [2, 3] although some interaction checkers highlight it [12, 13, 14]. This interaction has been scarcely studied, but other studies showed that the concomitant use of cyclophosphamide and hydrochlorothiazide did not imply a higher risk of agranulocytosis or chemotherapy discontinuation [15].

Given the limited evidence, we conducted this study to better define the interaction between thiazides and fluoropyrimidines. The primary objective was to assess if patients concomitantly treated present a higher risk of myelotoxicity. Secondary objectives were to evaluate the clinical relevance of this myelotoxicity, identify patients at higher risk of developing myelotoxicity, and assess the safety profile of this interaction.

This observational study has been reported following the STROBE [16] statement and ICMJE recommendations [17].

## 2 Material and Methods

### 2.1 Study Design and Setting

A retrospective cohort study based on electronic medical records was chosen. Patients were identified from the pharmacogenetics databases of the Clinical Pharmacology Department (CPD) and treated at a tertiary hospital in Madrid (Spain) from December 2013 to June 2024. Data extraction was performed from June to November 2024.

### 2.2 Participants

Adult patients diagnosed with colorectal cancer and treated with capecitabine or capecitabine‐oxaliplatin (CAPEOX) as a first‐ or second‐line treatment were included. Concomitant radiotherapy treatment was prohibited but previous was allowed. Patients were electronically followed from treatment beginning up to one month post‐treatment, loss of follow‐up, or cut‐off date (22nd of November 2024), whatever came first.

### 2.3 Variables

At the beginning potential confounders were identified, including other myelotoxic drugs, chemotherapy regimen, DPYD status, and relevant comorbidities. All performed hemograms were registered. A myelotoxicity episode was defined by any of the following: Hemoglobin < 12 g/dL, leucocytes < 4.00 × 10^3^/microL, neutrophils < 1.5 × 10^3^/microL, lymphocytes < 1.2 × 10^3^/microL, or platelets < 150 × 10^3^/microL. Since some patients may have had values considered as myelotoxic before initiating chemotherapy, the mean change of all previously mentioned parameters during follow‐up were calculated to evaluate myelotoxicity more accurately. To assess the safety profile, biochemistry blood parameters were documented. All AEs registered in the patients' medical histories were recorded, and some adverse events of special interest (AESI) were selected, including mucositis, palmar‐plantar erythrodysesthesia syndrome (PPES), acute kidney injuries (AKI), infections and bleeding episodes. Severe AEs (SAEs) were defined according to standard regulatory criteria and registered. Disease progression was assessed up to the cut‐off date.

### 2.4 Data Sources

All data came from our center information system SERVOLAB for laboratory values and SELENE for all the other information. Thiazide exposition was confirmed by the medication conciliation performed by the oncology department and electronic prescription system of the Community of Madrid. All data sources were consistent across cohorts to ensure data quality.

### 2.5 Methods to Prevent Bias

Only first or second‐line treatment treated with CAPE or CAPEOX regimes for colorectal cancer were included to avoid confounders of myelotoxicity.

### 2.6 Statistical Methods

Statistical analysis was performed with IBM SPSS (Statistical Package for the Social Sciences), version 21 (IBM Corporation, Somers, NY, USA) and RStudio (RStudio: Integrated Development Environment for R. Posit Software, PBC, Boston, MA). A significance level of 0.05 was applied. Descriptive analysis was realized expressing quantitative variables as mean (SD) or median (IQR), as appropriate, and categorical variables as frequency and percentage. Relative risk (RR) was calculated for myelotoxicity episodes between cohorts. Differences from baseline values between the two cohorts were calculated and compared using Student's *t*‐test or the Mann–Whitney U test, as appropriate. Chemotherapy changes and safety were compared between cohorts by RR. A multivariable Poisson regression model was also used to adjust relative risk estimates for potential confounding variables associated with myelotoxicity. Logistic regression and ANOVA analysis were used to identify predictors of myelotoxicity. To assess robustness, myelotoxicity was evaluated both as incidence and across predefined time windows (< 1 month, 1–3 months, 3–6 months, > 6 months). In addition, mean changes from baseline hematological values were analyzed to account for baseline cytopenias. Subgroup analyses were performed within CAPEOX‐treated patients to explore the potential influence of oxaliplatin. For multivariable logistic regression, Firth penalization was applied to reduce small‐sample bias. For the missing data, no imputation was performed.

### 2.7 Ethics

This study was performed following the Helsinki Declaration and approved by the local ethics committee (Hospital Universitario Puerta de Hierro Majadahonda; Act *n*°7/2024; approval code: 66/24). The protocol was submitted at the Spanish Clinical Studies Registry (ID: 0050–2024‐OBS) and the EMA‐RWD Catalogs (ID: 1000000181).

## 3 Results

**FIGURE 1:**
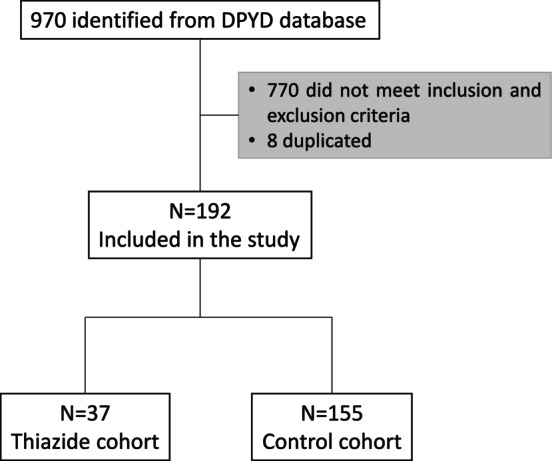
Flow diagram of patients. Flow diagram of the patients participating in the study.

970 patients were identified from the pharmacogenetics database of the CPD. 8 were duplicated. After checking the inclusion and exclusion criteria, the total number of patients was reduced to 192. Of those, 37 were classified in the thiazide cohort and 155 in the thiazide‐free cohort named control cohort (see Figure 1).

Relevant participants' characteristics are shown in Table 1. Full demographic results are presented in the (Tables S1–S3). The median follow‐up time in both cohorts was 126 days (IQR: 84–158), 104 days (IQR: 77–149) for the thiazide cohort and 146 days (IQR: 81–161) for the control cohort.

**TABLE 1:** Baseline demographic characteristics of study patients.

	Thiazide cohort *N* = 37	Control cohort *N* = 155	*p*	Total *N* = 192
Age (years), Median (IQR)	73 (67.5–78)	70 (60–76)	0.045	71 (62–77)
Sex, *N* (%)				
Male	23 (62.2)	95 (61.3)	0.992	118 (61.5)
Female	14 (37.8)	60 (38.7)		74 (38.5)
Comorbidities, *N* (%)				
Chronic kidney disease	2 (5.4)	3 (1.9)	0.234	5 (2.6)
Hepatic disease	0	8 (5.2)	0.158	8 (4.2)
Autoimmune disease	0	6 (3.9)	0.224	6 (3.1)
Cardiovascular disease	11 (29.7)	34 (21.9)	0.315	45 (23.4)
DPYD Function, *N* (%)				
Normal metabolizer	36 (97.3)	147 (94.8)	0.738	183 (95.3)
Intermediate metabolizer	1 (2.7)	8 (3.9)		9 (4.7)
Metamizole concomitant prescription, *N* (%)				
Yes	2 (5.4)	15 (9.7)	0.411	17 (8.9)
No	35 (94.6)	140 (90.3)		175 (91.1)
Concomitant prescription of other drugs that may cause myelotoxicity, *N* (%)				
Yes	10 (27.0)	55 (35.5)	0.329	65 (33.9)
No	27 (73.0)	100 (64.5)		127 (66.1)
Stage, *N* (%)				
I	0	0	0.190	0
II	6 (16.2)	45 (29.0)		51 (26.7)
III	29 (78.4)	92 (59.4)		121 (63.0)
IV	2 (5.4)	18 (11.6)		20 (10.4)
Hepatic metastases, *N* (%)	1 (2.7)	13 (8.4)	0.232	14 (7.3)
Bone marrow infiltration, *N* (%)	0	1 (0.6)	0.624	1 (0.5)
Number of patients in second line treatment, *N* (%)	3 (8.1)	15 (9.7)	0.769	18 (9.4)
Chemotherapy scheme, *N* (%)				
Capecitabine	20 (54.1)	78 (50.3)	0.683	98 (51.0)
Capecitabine‐Oxaliplatin	17 (45.9)	77 (49.7)		94 (49.0)
Capecitabine dose (mg/m^2^), Mean (SD)	937.8 (188.5)	921.5 (173.2)*	0.613	924.6 (175.9)*
Treatment intention, *N* (%)				
Adjuvant	33 (89.2)	139 (89.7)	0.524	172 (89.6)
Neoadjuvant	3 (8.1)	7 (4.5)		10 (5.2)
Palliative	1 (2.7)	9 (5.8)		10 (5.2)
Number of cycles, Mean (SD)	5.8 (2.6)	6.3 (2.6)	0.354	6.2 (2.6)

Abbreviations: %, percentage; DPYD, Dihydropyrimidine dehydrogenase; *N*, Number of patients; SD, Standard deviation.

* One patient is missing.

In the thiazide cohort 36 patients had hydrochlorothiazide prescribed and 1 indapamide. Median daily dosage of hydrochlorothiazide was 12.5 mg (IQR 12.5–25). The median time of thiazide treatment up to initiation of chemotherapy was 88 months (IQR 47–120).

During the follow‐up period, 31 patients (83.8%) in the thiazide cohort experienced at least one episode of myelotoxicity, compared to 117 patients (75.5%) in the control group. However, this difference was not statistically significant (RR: 1.11 [95% CI 0.94–1.31]; *p* = 0.280). The adjusted RR by other confounding factors that may cause myelotoxicity (Adjusted for oxaliplatin use, DPYD intermediate metabolizer, Metamizole concomitant use, other drugs that may cause myelotoxicity concomitant use, age > 70 years, CKD, second line of treatment, > 8 cycles, previous radiotherapy use.) was 1.09 [95% CI 0.71–1.64]. Myelotoxicity episodes were assessed during different periods (< 1, 1–3, 3–6, and > 6 months) and no differences were observed (see Table 2).

**TABLE 2:** Episodes of myelotoxicity on different periods of time.

Myelotoxicity episodes	Thiazide cohort *N* = 37	Control cohort *N* = 155	*p*	TOTAL
Baseline, *n*/*N* (%)	14/37 (37.8)	59/155 (38.1)	0.98	73/192 (38.0)
< 1 month, *n*/*N* (%)	19/36 (52.8)	64/151 (42.4)	0.259	83/187 (44.4)
1–3 months, *n*/*N* (%)	28/37 (75.7)	95/154 (61.7)	0.111	123/191 (64.4)
3–6 months, *n*/*N* (%)	21/25 (84.0)	70/105 (66.7)	0.089	91/130 (70.0)
> 6 months, *n*/*N* (%)	1/1 (100.0)	8/15 (53.3)	0.362	9/16 (56.3)

*Note:* *n* = Number of patients who presented myelotoxicity at that period. *N* = Total number of patients assessed at that period.

**FIGURE 2:**
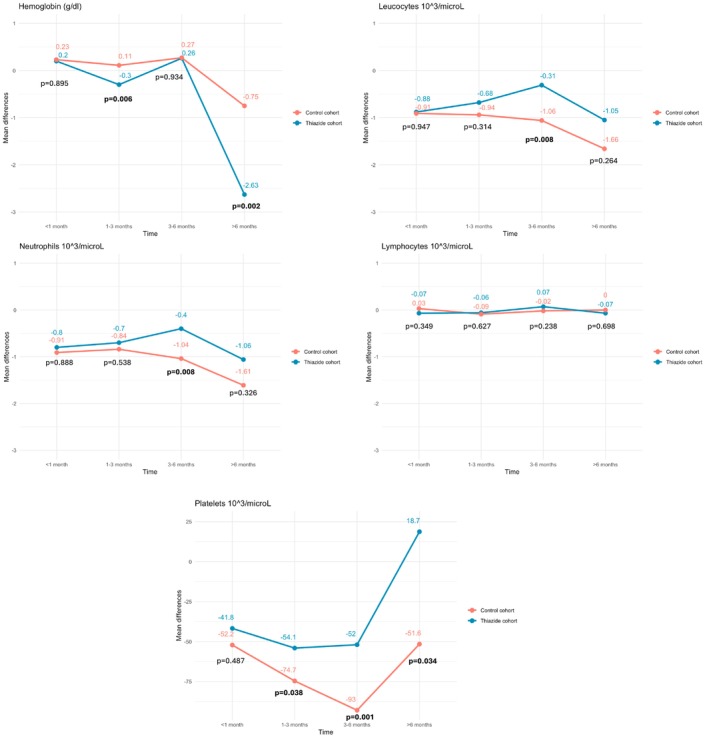
Mean variations from baseline values. Mean differences of different blood parameters to assess myelotoxicity between thiazide and thiazide‐free cohorts.

Since some patients already had values classified as myelotoxic before starting chemotherapy, mean variations from baseline values were compared between cohorts during different time points (< 1, 1–3, 3–6, and > 6 months). Only hemoglobin showed a statistically significant decrease in the thiazide cohort at the 1–3 months period (−0.3 g/dL vs. +0.11 g/dL; *p* = 0.006) and beyond 6 months (−2.63 g/dL vs. −0.75 g/dL; *p* = 0.002). Conversely, leukocytes, neutrophils, and platelets showed a greater statistically significant decrease in the control cohort (see Figure 2).

**FIGURE 3:**
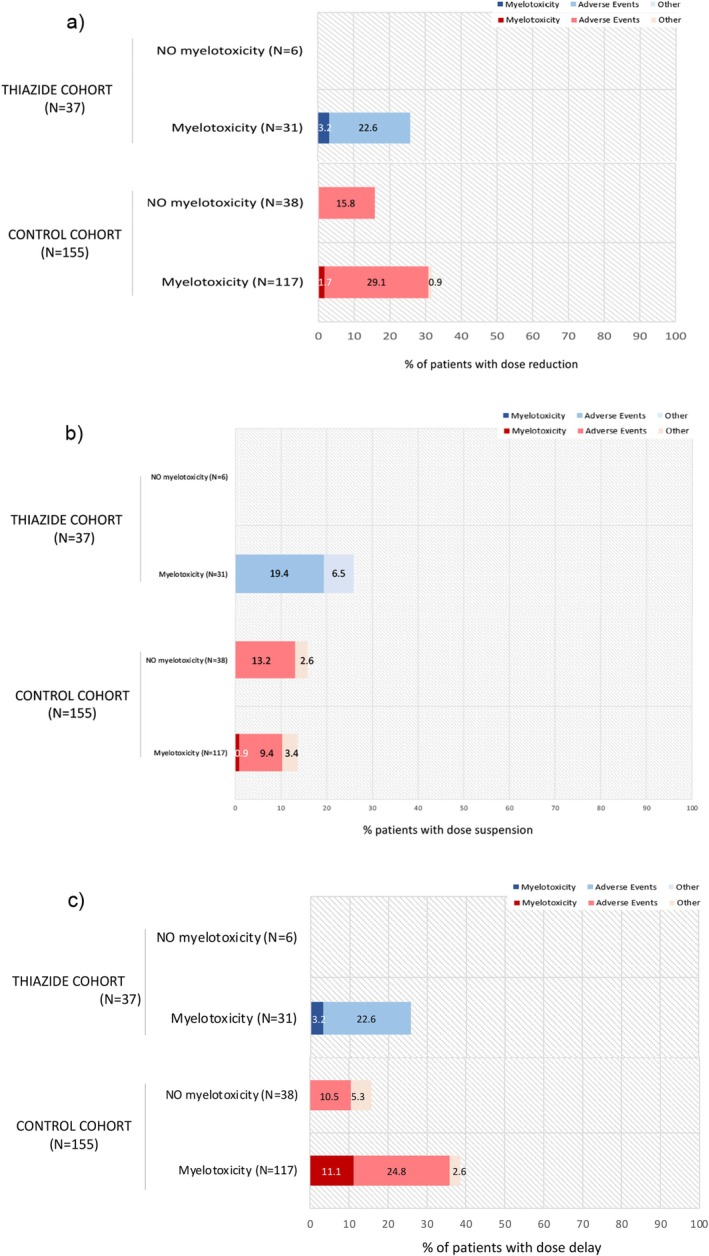
Patients capecitabine modifications. Percentage of patients with at least one modification of capecitabine during their chemotherapy period and its causes. (a) Percentage of patients with at least one dose reduction of capecitabine during their chemotherapy period. (b) Percentage of patients with dose suspension of capecitabine during their chemotherapy period. (c) Percentage of patients with at least one dose delay of capecitabine during their chemotherapy period. *N* = Number of patients.

The clinical significance of myelotoxicity was analyzed by studying dose modifications of capecitabine during the follow‐up. These modifications included dose reduction, dose suspension, and dose delay. The motive of each modification was registered, compared, and analyzed (see Figure 3).

Patients who developed myelotoxicity had more capecitabine modifications than those who did not. However, the overall need for treatment adjustments (including reduction, suspension or delay) did not differ between cohorts (RR = 0.87 [95% CI 0.58–1.30; *p* = 0.482]). Full capecitabine dose modifications results are available in the (Table S4).

No differences in disease progression were found between cohorts (RR = 0.60 [95% CI 0.23–1.42; *p* = 0.222]). In the thiazide cohort, patients with myelotoxicity did not present a higher risk of disease progression (RR = 1.29 [95% CI 0.173–9.634; *p* = 0.805]).

When predicting patients at higher risk of developing myelotoxicity, the univariable and multivariable logistic regression analysis did not show any potential risk factor in the thiazide cohort. In the control group, only the CAPEOX regimen was associated with an enhanced risk of myelotoxicity in the univariable and multivariable analysis (Univariable: OR = 2.32 [95% CI 1.08–4.98; *p* = 0.030], Multivariable: OR = 2.22 [95% CI 1.03–4.99; *p* = 0.04]).

In a prespecified robustness analysis restricted to CAPEOX‐treated patients, thiazide use was not associated with increased myelotoxicity. Given the higher myelotoxicity risk observed with CAPEOX (possibly attributable to oxaliplatin), we performed a subgroup analysis within CAPEOX‐treated patients, which showed no increased risk with concomitant thiazide use (RR = 0.92 [95% CI 0.69–1.22]; *p* = 0.767). Full logistic regression results are available in the (Tables S5 and S6).

ANOVA analysis revealed that patients with myelotoxicity had lower basal hemoglobin values compared to those without myelotoxicity in both cohorts: 12.87 g/dL (SD 1.35) vs. 14.38 g/dL (SD 1.79) [*p* = 0.022] in the thiazide cohort and 13.30 g/dL (SD 1.55) vs. 13.89 g/dL (SD 1.17) [*p* = 0.035] in control. In both cohorts, capecitabine dosage (mg/m^2^), baseline leucocytes, neutrophils, and platelets counts were not associated with a higher risk of presenting an episode of myelotoxicity. In the control group, lower baseline lymphocyte counts were linked to myelotoxicity (1.60 × 10^3^/microL [SD 0.61] group versus 1.93 × 10^3^/microL[SD 0.56]; *p* = 0.003). Full ANOVA results are available in the (Tables S7 and S8).

AEs by MedDRA System Organ Class (SOC) were similar between groups. These SOC were gastrointestinal disorders, general disorders and administration site conditions, nervous system disorders, skin, and subcutaneous tissue disorders, investigations and other AEs not classified in previous SOC. Most frequent AEs (> 20%) were nausea (58%), peripheral neurotoxicity (45%), diarrhea (41%), vomiting (38%), abdominal pain (34%), hypocalcemia (34%), asthenia (32%), constipation (22%), hypokalemia (22%), anorexia (20%). In the thiazide group, more patients showed elevated creatinine and bilirubin, while liver enzyme elevations (ALT, AST, GGT) were more common in the control group. There were no observed differences in the number of patients with AESI or SAEs (see Table 3).

**TABLE 3:** Safety Results. Number of episodes of adverse events registered. Number of patients with at least one analytical alteration.

Number of episodes of AE	Thiazide cohort *N* = 37	Control cohort *N* = 155	RR (95% CI) *p*
Gastrointestinal disorders, *N* (%)	26 (70.3)	103 (66.5)	1.06 (0.83–1.34); *p* = 0.657
General disorders and administration site conditions, *N* (%)	15 (40.5)	60 (38.7)	1.05 (0.68–1.62) *p* = 0.837
Nervous system disorders, *N* (%)	23 (62.2)	76 (49.0)	1.27 (0.94–1.71) *p* = 0.151
Skin and subcutaneous tissue disorders, *N* (%)	11 (29.7)	26 (16.8)	1.77 (0.97–3.25) *p* = 0.073
Investigations, *N* (%)	12 (32.4)	58 (37.4)	0.87 (0.52–1.44) *p* = 0.571
Other SOC, *N* (%)	15 (40.5)	56 (36.1)	1.12 (0.72–1.74) *p* = 0.617
Febrile Neutropenia, *N* (%)	0	0	NA
Bleeding, *N* (%)	0	5 (3.2)	NA
Oral mucositis, *N* (%)	3 (8.1)	10 (6.5)	1.26 (0.36–4.34) *p* = 0.719
Palmar‐Plantar Erythrodysesthesia Syndrome, *N* (%)	9 (24.3)	47 (30.3)	0.80 (0.43–1.49) *p* = 0.471
Acute Kidney Injury, *N* (%)	2 (5.4)	2 (1.3)	4.2 (0.6–28.7) *p* = 0.115
Infection, *N* (%)	6 (16.2)	33 (21.3)	0.76 (0.34–1.68) *p* = 0.491
AE that led to death, *N* (%)	0	0	NA
AE that was life‐threatening, *N* (%)	1 (2.7)	5 (3.2)	0.84 (0.10–6.96) *p* = 0.869
AE that caused permanent incapacity, *N* (%)	0	0	NA
AE that caused a hospitalization or has prolonged it, *N* (%)	2 (5.4)	13 (8.4)	0.64 (0.15–2.73) *p* = 0.544
AE that caused cancer or congenital anomaly, *N* (%)	0	0	NA
AE caused by an overdose, *N* (%)	0	0	NA
Number of patients with at least one analytical alteration			
Creatinine increased, *N* (%)	12 (32.4)	30 (19.4)	1.71 (0.94–3.12) *p* = 0.084
ALT increased, *N* (%)	10 (27.0)	72 (46.5)	0.58 (0.33–1.01) *p* = 0.032
AST increased, *N* (%)	11 (29.7)	65 (41.9)	0.71 (0.42–1.20) *p* = 0.173
GGT increased, *N* (%)	7 (18.9)	57 (36.8)	0.51 (0.26–1.03) *p* = 0.038
Bilirubin increased, *N* (%)	19 (51.4)	60 (38.7)	1.33 (0.92–1.92) *p* = 0.160

## 4 Discussion

Our study included a total of 192 patients, 37 in the thiazide cohort and 155 in the control cohort. Baseline characteristics were largely comparable between groups; however, patients in the thiazide cohort were slightly older than controls. Minor differences were also observed in the prevalence of autoimmune and hepatic disease in the control cohort and in the proportion of stage III disease in the thiazide cohort. Follow‐up was moderately longer in the control group. We acknowledge that the baseline age difference may represent a source of residual confounding inherent to the retrospective design, although no clinically meaningful imbalance was observed in treatment‐related variables.

The primary objective was not met, since no statistical differences were observed in the myelotoxicity incidence between cohorts. To address bias from baseline cytopenias, changes from initial hematological values were also analyzed to more accurately assess myelotoxicity risk. Only hemoglobin showed a statistically significant decrease, at the period between 1 to 3 months (−0.6 g/dL) and > 6 months (−2.63 g/dL) in the thiazide cohort. However, the clinical relevance appears limited, especially in the 1–3 months period. Other hematological parameters did not present relevant changes. Platelets and neutrophils showed a greater decrease in the control cohort. Secondary objectives were also not met. The clinical significance of this interaction was not considered relevant: the thiazide group had no higher treatment modifications, progression, or toxicity. Most of capecitabine changes were due to other AEs unrelated to myelotoxicity. No risk factors were identified for developing myelotoxicity in the thiazide cohort, except for having a lower basal level of hemoglobin, a risk factor also isolated in the control cohort. Oxaliplatin was identified as a risk factor in the control cohort. Therefore, CAPEOX is better interpreted as a regimen‐related factor rather than a confounder. Nevertheless, this is probably a spurious finding due to the limited number of patients included in the thiazide cohort (*n* = 37), since the decrease of hematological parameters is very common (< 1/10 patients) when oxaliplatin is used [18]. After these results, it was assessed whether patients treated with CAPEOX had a higher risk of myelotoxicity when thiazides were used concomitantly, and no association was found.

No differences were found in the safety profile, except for liver and kidney parameters, likely due to the baseline conditions of the patients.

Regarding limitations, retrospective designs carry inherent biases and possible inaccuracies due to missing or unrecorded data. Laboratory and prescription records were reliably extracted, but clinical AEs were only recorded if the physician registered them. Thus, these studies may result in residual confounding since not all confounding variables might have been collected and analyzed.

Secondly, this was a single‐center study conducted in a tertiary hospital in Madrid, which may limit the generalizability of our findings to other institutions and healthcare settings. Patients treated with 5‐FU monotherapy were not included, as it is typically combined with other myelotoxic agents, potentially masking specific fluoropyrimidine‐related toxicity. Hence, only capecitabine or CAPEOX regimens were included to minimize confounding. External validity may also be limited due to the low number of stage IV patients, as these are often treated with FOLFOX at our center instead of CAPEOX, improving internal consistency.

Thirdly, the reduced size of the thiazide cohort may have reduced the power of the study to detect differences, especially when comparing AEs or in the logistic regression analyses. This may explain the spurious result when no association was found between oxaliplatin use and a higher risk of myelotoxicity in the thiazide cohort. Although these complementary analyses were consistent, additional multicenter studies with larger thiazide‐exposed cohorts are needed to further confirm the robustness of the findings.

Overall, we found no evidence that the concomitant use of capecitabine and thiazides increases the risk of myelotoxicity. These results differ from the ones concluded by Leo E. Orr in 1981 [11] who reported an increased risk of myelotoxicity when thiazides were combined with cyclophosphamide, methotrexate, and 5‐FU, affecting posteriorly treatment management and presenting an increased risk of infections. Our results concur more with the ones presented by Hsu et al. where no association between the concomitant use of hydrochlorothiazide and cyclophosphamide and the number of chemotherapy‐related events was found [15].

Despite the limited evidence base for this interaction, due to the study conducted in 1981 [11] which was a single‐center study of 14 patients, and each patient was its own control, this interaction is still considered relevant in European 5‐FU SmPC [1] and drug interaction checkers [12, 13, 14]. Additionally, it has not been established which biological mechanism could explain this drug interaction. The major risk of granulocytopenia found in 1981 could be a spurious correlation, since no alteration in other hematological cell lines was found, whereas our study found a mild hemoglobin drop without broader cytopenias, but with an uncertain clinical implication. Our findings suggest that the clinical impact of this interaction may be lower than currently assumed; however, confirmation in larger multicenter real‐world studies is warranted before considering any changes to drug‐interaction resources. For that reason, when performing therapeutic individualization, the participation of expert physicians, such as clinical pharmacologists, can play a key role in evaluating drug interactions and their potential clinical relevance, as it is demonstrated in this case.

This study included patients treated under real‐world conditions. Consequently, the population included is representative of the standard clinical practice. The slight restriction in the inclusion criteria was performed to identify properly the assessment caused by the fluoropyrimidines with or without the interaction of thiazides. Since capecitabine is a prodrug of 5‐FU, these results could be extrapolated to 5‐FU patients as well.

## 5 Conclusion

Our study found no significant association between thiazide use and an increased risk of myelotoxicity or clinically relevant adverse events in colorectal cancer patients treated with capecitabine‐based regimens. These findings suggest that the clinical impact of this interaction may be lower than currently assumed; however, given the observational single‐center design and the limited size of the thiazide‐exposed cohort, confirmation in larger multicenter studies is warranted. To our knowledge, this is the first study to reassess the interaction of fluoropyrimidines and thiazides since 1981.

## Author Contributions

**Gerard Ronda‐Roca:** conceptualization (equal), formal analysis (equal), investigation (equal), writing – original draft (lead). **José Porcel‐Maleno:** investigation (equal). **Mónica Lobo‐Palomar:** investigation (equal). **Gustavo Centeno‐Soto:** methodology (equal), supervision (equal), writing – review and editing (equal). **Ana Ruiz‐Casado:** methodology (equal), supervision (equal), writing – review and editing (equal). **Belén Ruiz‐Antorán:** formal analysis (equal), methodology (equal), project administration (lead), supervision (equal), writing – review and editing (equal).

## Funding

The authors have nothing to report.

## Ethics Statement

The study was approved by a Research Ethics Committee for Medicinal Products (CEIm) and was conducted in accordance with the Declaration of Helsinki and Good Clinical Practice guidelines (CEIm Hospital Universitario Puerta de Hierro Majadahonda; Act *n*°7/2024; approval code: 66/24). The ethics committee waived the requirement for informed consent.

## Conflicts of Interest

The authors declare no conflicts of interest.

## Supporting information


**Table S1:** Full baseline characteristics.
**Table S2:** Baseline characteristics of neoplastic disease in the study population.
**Table S3:** Baseline characteristics of chemotherapy treatment in the study population.
**Table S4:** Capecitabine dose modifications during follow‐up and their causes.
**Table S5:** Univariate logistic regression analysis.
**Table S6:** Multivariate logistic regression analysis.
**Table S7:** ANOVA Results in thiazide cohort.
**Table S8:** ANOVA Results in control cohort.
*It represents the blood test results which are important results of our* study.

## Data Availability

The data that support the findings of this study are available from the corresponding author upon reasonable request.
