# Supplementary material for: Impact of Thiazides and Fluoropyrimidines Interaction on Myelotoxicity and Other Adverse Events in Real‐World Practice: A Retrospective Cohort Study
Source: Cancer Med. 2026 Feb 26;15(3):e71650. doi: 10.1002/cam4.71650 (PMC12946645; doi:10.1002/cam4.71650)
Supplement: Supplementary file 1 — Table S1: Full baseline characteristics. Table S2: Baseline characteristics of neoplastic disease in the study population. Table S3: Baseline characteristics of chemotherapy treatment in the study population. Table S4: Capecitabine dose modifications during follow‐up and their causes. Table S5: Univariate logistic regression analysis. Table S6: Multivariate logistic regression analysis. Table S7: ANOVA Results in thiazide cohort. Table S8: ANOVA Results in control cohort. It represents the blood test results which are important results of our study. [file CAM4-15-e71650-s001.docx]

**Supplementary Material**

1. **Supplementary Table S1.** Full baseline characteristics

|  | Thiazide cohort  N=37 | Control Cohort  N=155 | TOTAL  N=192 |
| --- | --- | --- | --- |
| Age (year), Mean (SD) | 72.9 (8.4) | 67.6 (13.8) | 68.6 (13.1) |
| Sex, male, N (%) | 23 (62.2) | 95 (61.3) | 118 (61.5) |
| High blood pressure, N (%) | 37 (100) | 61 (39.4) | 97 (50.5) |
| Dyslipidemia, N (%) | 19 (51.4) | 53 (34.2) | 72 (37.5) |
| Diabetes Mellitus, N (%) | 10 (27.0) | 23 (14.8) | 33 (17.2) |
| Hepatic disease, N (%) | 0 | 8 (5.2) | 8 (4.2) |
| Chronic kidney disease, N (%) | 2 (5.4) | 3 (1.9) | 5 (2.6) |
| Autoimmune disease, N (%) | 0 | 6 (3.9) | 6 (3.1) |
| Cardiovascular disease, N (%) | 11 (29.7) | 34 (21.9) | 45 (23.4) |
| Respiratory disease, N (%) | 7 (18.9) | 15 (9.7) | 22 (11.5) |
| DPYD Function, N (%) |  |  |  |
| Normal Metabolizer | 36 (97.3) | 147 (94.8) | 183 (95.3) |
| Intermediate metabolizer | 1 (2.7) | 8 (3.9) | 9 (4.7) |
| Metamizole, N (%) | 2 (5.4) | 15 (9.7) | 17 (8.9) |
| Antihistaminic, N (%) | 0 | 10 (6.5) | 10 (5.2) |
| Antimicrobials, N (%) | 0 | 3 (1.9) | 3 (1.6) |
| Immunosuppressors, N (%) | 2 (5.4) | 7 (4.5) | 9 (4.7) |
| Psychotropics, N (%) | 7 (18.9) | 23 (14.8) | 30 (15.6) |
| Antiseizures, N (%) | 0 | 1 (0.6) | 1 (0.5) |
| Antiarrhythmic, N (%) | 1 (2.7) | 19 (12.3) | 20 (10.4) |

|  | Thiazide cohort  N=37 | Control cohort  N=155 | TOTAL  N=192 |
| --- | --- | --- | --- |
| Neoplasy location, N (%) |  |  |  |
| Colorectal | 36 (97.3) | 152 (98.1) | 188 (97.9) |
| Canal anal | 1 (2.7) | 1 (0.6) | 2 (1.0) |
| Other* | 0 | 2 (1.3) | 2 (1.0) |
| Stage, N (%) |  |  |  |
| II | 6 (16.2) | 45 (29.0) | 51 (26.7) |
| III | 29 (78.4) | 92 (59.4) | 121 (63.0) |
| IV | 2 (5.4) | 18 (11.6) | 20 (10.4) |
| Hepatic metastases, N (%) | 1 (2.7) | 13 (8.4) | 14 (7.3) |
| Bone marrow infiltration, N (%) | 0 | 1 (0.6) | 1 (0.5) |
| Number of patients in second line treatment, N (%) | 3 (8.1) | 15 (9.7) | 18 (9.4) |
| Previous radiotherapy, N (%) | 7 (18.9) | 25 (16.1) | 32 (16.7) |
| Total Grays, Mean (SD) | 43.7 (20.1) | 40.7 (11.8) | 41.4 (13.6) |

1. Supplementary Table S2. Baseline characteristics of neoplastic disease in the study population
2. Supplementary Table S3. Baseline characteristics of chemotherapy treatment in the study population

|  | Thiazide cohort  N=37 | Control cohort  N=155 | Total  N=192 |
| --- | --- | --- | --- |
| Chemotherapy scheme |  |  |  |
| Capecitabine | 20 (54.1) | 78 (50.3) | 98 (51.0) |
| Capecitabine-Oxaliplatin | 17 (45.9) | 77 (49.7) | 94 (49.0) |
| Capecitabine dose/m^2^ Mean (SD) | 937.8 (188.5) | 921.5 (173.2) * | 924.6 (175.9) * |
| % Oxaliplatin total dose  Mean (SD) ** | 98.2 (7.3) | 95.8 (9.3) | 71.5 (43.0) |
| Treatment intention |  |  |  |
| Adjuvant | 33 (89.2) | 139 (89.7) | 172 (89.6) |
| Neoadjuvant | 3 (8.1) | 7 (4.5) | 10 (5.2) |
| Palliative | 1 (2.7) | 9 (5.8) | 10 (5.2) |
| Number of cycles  Mean (SD) | 5.8 (2.6) | 6.3 (2.6) | - 1. (2.6) |

|  | Dose reduction | | | | Dose suspension | | | | Dose delay | | | | Any change |
| --- | --- | --- | --- | --- | --- | --- | --- | --- | --- | --- | --- | --- | --- |
|  | Myelotox | Other AE | Other | Total redu | Myelotox | Other AE | Other | Total susp | Myelotox | Other AE | Other | Total delay |  |
| Thiazide N=37 | | | | | | | | | | | | | |
| Myelotoxicity N=31 | 1 (3.2) | 7 (22.6) | 0 | 8 (25.8) | 0 | 6 (19.4) | 2  (6.5) | 8 (25.8) | 1 (3.2) | 7 (22.6) | 0 | 8 (25.8) | **16 (51.6)** |
| NO Myelotoxicity N=6 | 0 | 0 | 0 | 0 | 0 | 0 | 0 | 0 | 0 | 0 | 0 | 0 | **0** |
| Total thiazide | 1 (2.7) | 7 (18.9) | 0 | 8 (21.6) | 0 | 6 (16.2) | 2  (5.4) | 8 (21.6) | 1 (2.7) | 7 (18.9) | 0 | 8 (21.6) | **16 (43.2)** |
| Control N=155 | | | | | | | | | | | | | |
| Myelotoxicity N=117 | 2 (1.7) | 34 (29.1) | 1  (0.9) | 37 (31.6) | 1  (0.9) | 11 (9.4) | 4  (3.4) | 16 (13.7) | 13 (11.1) | 29 (24.8) | 3 (2.6) | 45 (38.5) | **12 (31.6)** |
| NO Myelotoxicity N=38 | 0 | 6 (15.8) | 0 | 6 (15.8) | 0 | 5 (13.2) | 1  (2.6) | 6 (15.8) | 0 | 4 (10.5) | 2 (5.3) | 6 (15.8) | **65 (55.6)** |
| Total control | 2 (1.3) | 40 (25.8) | 1  (0.6) | 43 (27.7) | 1  (0.6) | 16 (10.3) | 5  (3.2) | 22 (14.2) | 13 (8.4) | 33 (21.3) | 5  (3.2) | 51 (32.9) | **77 (49.7)** |

1. Supplementary Table S4. Capecitabine dose modifications during follow-up and their causes
2. Supplementary Table S5. Univariate logistic regression analysis

|  | Thiazide cohort  N=37 | Control cohort  N=155 |
| --- | --- | --- |
| Sex (Men) | 0.28 (0.03-2.67); p=0.266 | 1.04 (0.49-2.21); p=0.911 |
| Age (>70 years) | 1.10 (0.17-7.00); p=0.920 | 1.46 (0.69-3.07); p=0.322 |
| Cardiovascular disease | 2.38 (0.25-23.17); p=0.455 | 0.88 (0.37-2.09); p=0.764 |
| Thiazide dose >25mg/day | 0.45 (0.08-2.68); p=0.380 | Not applicable |
| Metamizole | 6.00 (0.32-112.26); p=0.231 | 0.20 (0.03-1.57); p=0.125 |
| Other drugs | 0.70 (0.11-4.56); p=0.705 | 1.48 (0.67-3.28); p=0.334 |
| DPYD Intermediate metabolizer | Not calculable due to small number of patients | 5.00 (0.15-166.59); p=0.368 |
| 2^nd^ line treatment | Not calculable due to small number of patients | 0.44 (0.1-2.1); p=0.301 |
| Previous Radiotherapy | 1.2 (0.12-12.3); p=0.878 | 2.70 (0.76-9.59); p=0.124 |
| Capecitabine-Oxaliplatin | 0.36 (0.06-2.28) | 2.32 (1.08-4.98); p=0.030 |
| 4-8 cycles | 3.00 (0.47-19.04); p=0.244 | 1.91 (0.89-4.09); p=0.096 |
| >8 cycles | Not calculable due to small number of patients | 0.46 (0.03-7.79); p=0.590 |

|  | Thiazide cohort  N=37 | Control cohort  N=155 | Total  N=192 |
| --- | --- | --- | --- |
| Sex (Men) | 1.68 (0.31-9.62);  p=0.540 | 0.84 (1.05-6.78); p=0.654 | 0.96 (0.47-1.94); p=0.920 |
| Age (>70 years) | 0.99 (0.11-8.46);  p=0.997 | 0.63 (0.27-1.41); p=0.258 | 0.68 (0.33-1.41); p=0.306 |
| Cardiovascular disease | 1.52 (0.19-31.06);  p=0.718 | 0.76 (0.32-1.86); p=0.537 | 0.75 (0.34-1.71); p=0.487 |
| Thiazide dose >25mg/day | 0.45 (0.06-2.72);  p=0.378 | Not applicable | Not applicable |
| Metamizole | 0.36 (0.02-4.69);  p=0.435 | 1.04 (0.20-7.07); p=0.964 | 0.75 (0.20-3.07); p=0.671 |
| Other drugs | 0.94 (0.12-10.14);  p=0.952 | 1.01 (0.43-2.48); p=0.984 | 0.88 (0.40-2.02); p=0.760 |
| DPYD Intermediate metabolizer | Not calculable due to small number of patients | 5.35 (0.59-708.37); p=0.159 | 7.07 (0.84-927.32); p=0.08 |
| 2^nd^ line treatment | Not calculable due to small number of patients | 0.89 (0.22-4.06); p=0.875 | 0.70 (0.20-2.65); p=0.583 |
| Previous Radiotherapy | 3.71 (0.11-748.42); p=0.487 | 0.74 (0.25-2.30); p=0.594 | 0.93 (0.33-2.77); p=0.844 |
| Capecitabine-Oxaliplatin | 0.54 (0.08-3.06);  p=0.479 | 2.23 (1.03-5.00);  p=0.04 | 1.76 (0.88-3.62); p=0.113 |
| 4-8 cycles | 0.88 (0.08-7.13);  p=0.906 | 1.42 (0.62-3.25); p=0.398 | 1.31 (0.61-2.80); p=0.481 |
| >8 cycles | Not calculable due to small number of patients | 0.69 (0.07-9.81); p=0.760 | 0.59 (0.06-7.82); p=0.655 |

1. Supplementary Table S6. Multivariate logistic regression analysis

Note: Multivariate logistic regression was calculated applying the Firth penalization

| Dependent variable | Myelotoxicity | No Myelotoxicity | P value |
| --- | --- | --- | --- |
| Capecitabine dosage (mg/m2) | 890.83 (187.40) | 946.90 (190.41) | 0.512 |
| Baseline Hemoglobin (mg/dl) | 12.87 (1.35) | 14.38 (1.79) | 0.022 |
| Baseline Leucocytes (10^3^/microL) | 6.69 (1.63) | 7.97 (1.18) | 0.078 |
| Baseline Neutrophils (10^3^/microL) | 4.36 (1.54) | 4.90 (0.88) | 0.411 |
| Baseline Lymphocytes (10^3^/microL) | 1.63 (0.74) | 2.10 (0.29) | 0.136 |
| Baseline Platelets (10^3^/microL) | 235.71 (72.83) | 289.50 (47.29) | 0.093 |

1. Supplementary Table S7. ANOVA Results in thiazide cohort
2. Supplementary Table S8. ANOVA Results in control cohort

| Dependent variable | Myelotoxicity | No Myelotoxicity | P value |
| --- | --- | --- | --- |
| Capecitabine dosage (mg/m2) | 929.72 (174.26) | 896.26 (169.68) | 0.303 |
| Baseline Hemoglobin (mg/dl) | 13.30 (1.55) | 13.89 (1.17) | 0.035 |
| Baseline Leucocytes (10^3^/microL) | 6.83 (2.06) | 7.13 (1.91) | 0.434 |
| Baseline Neutrophils (10^3^/microL) | 4.43 (1.89) | 4.31 (1.64) | 0.742 |
| Baseline Lymphocytes (10^3^/microL) | 1.60 (0.61) | 1.93 (0.56) | 0.003 |
| Baseline Platelets (10^3^/microL) | 274.77 (109.02) | 268.24 (68.28) | 0.303 |
